# Supplementary material for: Abnormal expression and the significant prognostic value of aquaporins in clear cell renal cell carcinoma
Source: PLoS One. 2022 Mar 4;17(3):e0264553. doi: 10.1371/journal.pone.0264553 (PMC8896691; doi:10.1371/journal.pone.0264553)
Supplement: Attachment — Submitted filename: Response to reviewers.docx [file pone.0264553.s001.docx]

Dear Editors and Reviewers,

Thank you very much for your comments and support. We have revised the manuscript and resubmitted it. Point by point responses to the reviewers’ comments are listed below this letter. Correspondingly, the modifications will also be presented in the revised manuscript. We look forward to hearing from you soon. We also would like to express our sincere thanks to the reviewers and editors for the constructive and positive comments.

**Editorial Board Member comments**

**Comments 1:** Please ensure that your manuscript meets PLOS ONE's style requirements, including those for file naming.

**Answer 1:** Thank you very much for your reminders and suggestions. We apologize for the inconvenience caused to you. We have re-checked the manuscript format to make it meet the submission requirements of plos one.

**Comments 2:** We note that Figure 3 in your submission contain copyrighted images. All PLOS content is published under the Creative Commons Attribution License (CC BY 4.0), which means that the manuscript, images, and Supporting Information files will be freely available online, and any third party is permitted to access, download, copy, distribute, and use these materials in any way, even commercially, with proper attribution.

**Answer 2:** This is a very important reminder. We were aware of this copyright issue and immediately contacted the original copyright holder. We have obtained written permission from the copyright owner to publish these figures exclusively under the CC BY 4.0 license. The Content Permission Form has been submitted as an “other” file. The legend of Figure 3 has also been modified as required.

**Reviewers' comments**

**Reviewer #1:**

**Comments 1:** My concern is about the difference in the expression of AQPs between normal kidneys ccRCC. For example, the expression of AQP9,10, 12A, and 12B in normal kidneys is still unknown and was not detected. However, the authors demonstrated their expression and the roles in the ccRCC (for example, lines: 195, 211, 214, 242, and 243). Importantly, the authors demonstrated the genetic mutation of AQPs in ccRCC and stated that enhanced mRNA expression is the most common change in these samples. Then, can we think that AQP9, 10, 12A are abundantly expressed in ccRCC and they have their own functions, which are not expressed in normal kidneys?

**Answer 1:** Very appreciate for your constructive comments. AQP9 is an important transmembrane protein. After analyses of the UALCAN and HPA, we found that the mRNA and protein expression level of AQP9 in normal kidney tissues and ccRCC tissues were significantly different. AQP9 plays a role in glycerol transport and participates in the regulation of cellular inflammation. It can regulate a series of inflammation-related signal pathways, such as IL6/JAK-STAT3, IL2-STAT5 and TNFα signal pathways, among which the pathway, IL-6/JAK-STAT3, is abnormally activated in many cancers and is usually associated with poor clinical prognosis [1]. In the tumor microenvironment, the IL-6/JAK-STAT3 signaling pathway promotes tumor cell proliferation, invasion and metastasis, while strongly inhibiting tumor immune response [2].

Studies of AQP10 in intestinal epithelial cells are relatively comprehensive. In addition to transporting water, AQP10 is also involved in the penetration of ammonia, erythritol and metalloids, including toxic inorganic arsenic and glycerol [3, 4]. Therefore, AQP10 may play a broader unknown role than water transportation. Recent reports on the expression of AQP10 in the stratum corneum and adipocytes of the skin indicated that AQP10 might be widely expressed. However, their western blot showed that there were several AQP10 bands without monomeric bands, in contrast to the clear expression of AQP3 in the skin. This indicated that the expression of AQP10 was the lowest [5]. There was no significant difference in the expression level of AQP10 in normal kidney tissues and ccRCC tissues. The TCGA data showed that compared with other members, the expression level of AQP10 was lower, which is more consistent with its performance in other tissues.

AQP12A is a subtype of AQP12. Existing studies have shown that AQP12 is mainly expressed in pancreatic tissue, and the inactivation or mutation of AQP12 is closely related to the occurrence of pancreatitis [6]. According to the mRNA expression information in the TCGA database, AQP12A has been detected in some samples of normal kidney tissues and ccRCC tissues, but most of the samples are not detected. This shows that the relationship between AQP12A and kidney or kidney cancer is relatively weaker than that of other members of AQPs. At the same time, the protein expression level of AQP12A is low both in normal tissues and tumor tissues, and there is no significant change in the expression of AQP12A in different stages of ccRCC. The difference indicates that even though there are changes in gene amplification and gene deletion in gene mutation analysis, its role in ccRCC may not be significant.

In general, the difference in the expression level of AQP9 in normal kidney tissues and renal clear cell carcinoma and the related pathways involved in tumors are relatively clear; while AQP10/12A can only detect a certain degree of expression in some normal tissues. However, these members play a certain role in the development of other diseases, and the pathways involved may be related to the occurrence of ccRCC. This requires us to follow up and investigate the mechanism in depth.

**Comments 2:** The authors demonstrated the direct correlation between AQPs expression (AQP1 -12) and the infiltration of immune cells. Is there any background that shows a direct link between AQPs expression levels and the infiltration of immune cells?

**Answer 2:** It’s a very professional comment. At present, many studies have confirmed the relationship between AQPs and inflammation. First of all, regarding the expression of immune cells and AQPs, AQPs are expressed in human immune cells of the innate and adaptive immune system [7]. In human blood leukocytes, AQP1 and AQP9 were detected and upregulated after intravenous or in vitro lipopolysaccharide stimulation [8]. AQP9 is also increased in activated polymorphonuclear leukocytes in patients with systemic inflammatory response syndrome and infective endocarditis [9, 10]. Basic studies have found that AQP1, AQP3, and AQP5 are detected in tumor-infiltrating lymphocytes around lung cancer, and AQP3 and AQP5 are detected in dendritic cells. Their expression is related to the activation and proliferation of these immune cells [11].

The second is the cytokine and growth factor signaling pathways that AQPs participate in inflammation. In vitro experiments have shown that inhibition or silencing of AQP3 will partially block LPS initiation and reduce the production of interleukin IL-6, IL-1β and TNF-α, indicating that there is a connection between AQP3 function and TLR4 in the initiation of macrophages [12, 13]. Another study reports that AQP1 expression increases after LPS administration, while AQP5 mRNA expression decreases [14]. In addition, patients with systemic inflammatory response syndrome have increased AQP9 expression in neutrophils compared to healthy controls [9].

Finally, it is about the regulation of immune cells by AQPs. Several studies have shown that AQPs regulate the migration of different immune cells. For example, AQP3 is essential for T cell function and macrophage migration, while AQP5 and AQP9 regulate neutrophil migration and affect the survival of sepsis [15, 16].

In general, AQPs do play an important role in inflammation by regulating immune cell migration and participating in inflammation-related cytokine pathways. Studies have found the expression of AQPs in infiltrating immune cells in the tumor microenvironment of lung cancer. However, there is still a lack of relevant research on kidney cancer.

**Comments 3:** The authors demonstrated the prognostic value of AQPs in ccRCC, by examining the levels of mRNA expression of AQPs. In general, protein expression levels are affected by post-transcriptional and post-translational modification, and thus protein expression levels are not always the same as their mRNA levels. However, the proteins are mainly responsible for the function. Then how can we interpret the results of mRNA in the clinical setting? Please discuss this issue in the discussion section.

**Answer 3:** Thank you for your reminder, this is a valuable suggestion. What we consider is that if there is no change in mRNA expression, the possibility of protein change is very low, because the basis of the translation process is dependent on the transcription process [17, 18]. Therefore, mRNA expression changes can be used as a method to find DEGs preliminary screening, but as you said, further verification of protein expression is needed. Following your suggestion, we discussed this issue in the discussion section and marked it with the "Track Changes" function.

**Reviewer #2:**

**Comments 1:** The Abstract would be more informative for a broad audience if written with minimal use of acronyms. Abbreviations that will be kept in the Abstract must be defined. The Abstract as written is not sufficent in essential details; results ideally would be presented as concrete findings with specific major results, rather than vague statements. Phrases such as "The expression levels of different AQPs members in ccRCC had different trends..." or "This study aims to explore..." have no information content. The most informative approach is to specify the results by AQP class, to state the levels of up- or downregulation ,to report fold changes in risk factors, and to present interpretive summaries of the main findings.

**Answer 1:** It’s a very meaningful comment. We have provided a complete definition of the abbreviations in the abstract to ensure that readers can correctly understand the meaning of the abbreviations. Through your guidance, we have understood that the abstract is improperly written and there are many repetitions. We have revised the places you specifically pointed out, and also revised other parts of the abstract. The abstract part has been marked with the "Track Changes" function.

**Comments 2:** The Methods section briefly summarizes general methods used for data acquisition and analysis. Inclusion of an in-depth Supplementary file for the Methods is recommended highly, fully extending each of the Methods sections (Oncomine, ULACAN, TCGA, GEPIA, cBioPortal, GeneMANIA and other analyses) in order to provide the level of detail that would be necessary for independent replication and verification of the findings.

**Answer 2:** It’s a very constructive comment. We have supplemented the steps involved in the method section. The modified parts have been marked by the "Track Changes" function.

**Comments 3:** The Results section should be edited throughout to clarify the lines of argument and to incorporate substantive details with justifications. The overall aim of carrying out a multivariate analysis that draws from multiple databases is laudable, and the focus of this research project is important, but the results as presented have not yet been sufficiently analyzed or coherently integrated to achieve clear and convincing conclusions. As an example, starting just with the first section of the Results (beginning line 161) what precisely were the 20 tumors that were selected, why were only 20 used out of the many samples available in public domain archives, and how were these choices justified? Following on, the presentation of the findings comes across as a random mixture of new observations with previously published findings and thus lacks clarity, as illustrated by statements such as "the expression level of AQP6 was up-regulated or down-regulated in different test results, which was inconsistent. For AQP9/10/12A/12B, no test data could provide suitable results under the established screening conditions." The significance and interpretations of these observations are not clear, and the main points being made are not well articulated.

**Answer 3:** Very appreciate for your comments. In the first part of the results, we presented the results of the difference in mRNA expression of AQPs in pan-cancer in the Oncomine database. As a product adapted to the big data analysis environment, Oncomine brings together 715 data sets and 86733 samples of different cancers in the human body. Cancer categories are bladder cancer, brain and CNS cancer, breast cancer, cervical cancer, colorectal cancer, esophageal cancer, gastric cancer, head and neck cancer, kidney cancer, leukemia, liver cancer, lung cancer, lymphoma, melanoma, myeloma, ovarian cancer, pancreatic cancer, prostate cancer, sarcoma and other cancer. The Oncomine platform uses these as categories to visualize the mRNA expression changes of different human genes in pan-cancers (Fig 1), so we called it ‘in 20 different tumors’ in the article. At the same time, we wanted to show the visualization results of the changes in the expression of AQPs in different datasets of kidney cancer through Figure 1. The color of the square represented the change trend (red-up regulation, blue-down regulation), and the number represented datasets meeting the screening criteria (gene rank, 10%; fold change, 2; and P-value, 0.01). At the same time, we found that these datasets were not all related to ccRCC, so we screened these datasets one by one and made detailed statistics and data source references for the related datasets of ccRCC (Table 1). We described in the article that " the expression level of AQP6 was up-regulated or down-regulated in different test results, which was inconsistent. " was also the reason for the datasets. Among AQPs, only the expression differences of AQP6 had opposite trends in different datasets. After reading the dataset information related to AQP6, we found that the datasets showing the up-regulation of AQP6 expression in kidney cancer tissues were not the datasets related to ccRCC, and the datasets that obtained the expression down-regulation result were the ccRCC datasets. This result is consistent with the result of the subsequent UALCAN database analysis (Fig 2). And AQP9/10/12A/12B did not have any datasets that could meet the established screening conditions (gene rank, 10%; fold change, 2; and P-value, 0.01). If the screening conditions were lowered, any datasets that meet the conditions could be obtained, but this would result in a lack of diversity in the information obtained from other genes.

**Comments 4:** Unfortunately, the lack of clarity and logic persists throughout the Results section. For example, in Table 1, the evidence supporting the reported fold changes is not clearly explained. Information in the "Ref" column is not presented in a format that would allow a reader to find the cited work. Which cancers are being compared in this Table is not indicated.

**Answer 4:** Thank you for your reminding. Table 1 is a detailed summary of the datasets related to ccRCC in the results of the Oncomine database (see the header of Table 1). The fold changes of the difference between AQPs in ccRCC and normal kidney tissues are the real data obtained from the Oncomine database. The relevant documents involved in the ‘Ref’ column were listed in the text ‘Next, we analyzed the specific data (Table 1)[23],[24],[25],[26],[27], [28].’

**Comments 5:** Perhaps overall formatting has been altered inadvertently during document uploading, but the information shown for apparently more than one Table (?) on pages 18-27 seems incomprehensible.

**Answer 5:** Many thanks for your comments. A total of 6 tables are shown on pages 18-27. Among them, Table2 is the baseline data table of 526 cases of ccRCC samples, and Table3 is the result of univariate Cox regression analysis of AQPs, indicating that AQP0/1/4/9 are prognostic related factors. Table4-7 are the results of multivariate Cox regression analysis of AQP0/1/4/9, respectively, indicating that AQP1/9 are independent prognostic factors. We made a mistake by treating table titles as table legends and then writing table titles below tables, which caused you to be unable to understand the content of the table correctly. Now we have revised all the tables in the manuscript. The modified parts have been marked by the "Track Changes" function.

**Comments 6:** Image quality of the Figures is unacceptably low. Images are blurry and pixelated and should be replaced with higher resolution copies. Some (such as Fig 2) are illegible. Overall this area of work has promise, but has not yet been sufficiently or carefully developed.

**Answer 6:** Thank you for your reminding. We have re-made the picture, and set the resolution, file size, font, compression method and other aspects based on the picture guide of plos one.

**Comments 7:** References cited need be directly relevant to the text statements, and should acknowledge seminal work when possible. For example, the review by Castle 2005 (7) would not be the best reference for the reported classification of AQP12 subtypes (line 51). Equal care is needed for every citation. All references throughout the MS should be double-checked as the best choices for the matching statements of fact.

**Answer 7:** We have changed the wrong citation, replaced with "The role of mammalian superaquaporins inside the cell: An update" (PMID:33811846). We also checked the rationality of other documents again.

**Comments 8:** Correcting minor errors such as imprecise uses of some words, defining all acronyms at first use, and replacing colloquialisms are small but necessary details to be addressed throughout the text.

**Answer 8:** Many thanks for your recommendation. For imprecise uses of some words, defining all acronyms at first use, and replacing colloquialisms and other questions, we have reviewed the full text and made several revisions. The modified parts have been marked by the "Track Changes" function.

**References:**

1.	Xu WH, Shi SN, Xu Y, Wang J, Wang HK, Cao DL, et al. Prognostic implications of Aquaporin 9 expression in clear cell renal cell carcinoma. J Transl Med. 2019;17(1):363.

2.	Johnson DE, O'Keefe RA, Grandis JR. Targeting the IL-6/JAK/STAT3 signalling axis in cancer. Nat Rev Clin Oncol. 2018;15(4):234-48.

3.	Laforenza U, Scaffino MF, Gastaldi G. Aquaporin-10 represents an alternative pathway for glycerol efflux from human adipocytes. PLoS One. 2013;8(1):e54474.

4.	Litman T, Sogaard R, Zeuthen T. Ammonia and urea permeability of mammalian aquaporins. Handb Exp Pharmacol. 2009(190):327-58.

5.	Jungersted JM, Bomholt J, Bajraktari N, Hansen JS, Klaerke DA, Pedersen PA, et al. In vivo studies of aquaporins 3 and 10 in human stratum corneum. Arch Dermatol Res. 2013;305(8):699-704.

6.	Ishibashi K, Tanaka Y, Morishita Y. The role of mammalian superaquaporins inside the cell: An update. Biochim Biophys Acta Biomembr. 2021;1863(7):183617.

7.	Talwar S, Munson PJ, Barb J, Fiuza C, Cintron AP, Logun C, et al. Gene expression profiles of peripheral blood leukocytes after endotoxin challenge in humans. Physiol Genomics. 2006;25(2):203-15.

8.	Vassiliou AG, Maniatis NA, Orfanos SE, Mastora Z, Jahaj E, Paparountas T, et al. Induced expression and functional effects of aquaporin-1 in human leukocytes in sepsis. Crit Care. 2013;17(5):R199.

9.	Matsushima A, Ogura H, Koh T, Shimazu T, Sugimoto H. Enhanced expression of aquaporin 9 in activated polymorphonuclear leukocytes in patients with systemic inflammatory response syndrome. Shock. 2014;42(4):322-6.

10.	Thuny F, Textoris J, Amara AB, Filali AE, Capo C, Habib G, et al. The gene expression analysis of blood reveals S100A11 and AQP9 as potential biomarkers of infective endocarditis. PLoS One. 2012;7(2):e31490.

11.	Moon C, Rousseau R, Soria JC, Hoque MO, Lee J, Jang SJ, et al. Aquaporin expression in human lymphocytes and dendritic cells. Am J Hematol. 2004;75(3):128-33.

12.	da Silva IV, Cardoso C, Martinez-Banaclocha H, Casini A, Pelegrin P, Soveral G. Aquaporin-3 is involved in NLRP3-inflammasome activation contributing to the setting of inflammatory response. Cell Mol Life Sci. 2021;78(6):3073-85.

13.	Rabolli V, Wallemme L, Lo Re S, Uwambayinema F, Palmai-Pallag M, Thomassen L, et al. Critical role of aquaporins in interleukin 1beta (IL-1beta)-induced inflammation. J Biol Chem. 2014;289(20):13937-47.

14.	Rump K, Brendt P, Frey UH, Schafer ST, Siffert W, Peters J, et al. Aquaporin 1 and 5 expression evoked by the beta2 adrenoreceptor agonist terbutaline and lipopolysaccharide in mice and in the human monocytic cell line THP-1 is differentially regulated. Shock. 2013;40(5):430-6.

15.	Rump K, Adamzik M. Function of aquaporins in sepsis: a systematic review. Cell Biosci. 2018;8:10.

16.	Hara-Chikuma M, Chikuma S, Sugiyama Y, Kabashima K, Verkman AS, Inoue S, et al. Chemokine-dependent T cell migration requires aquaporin-3-mediated hydrogen peroxide uptake. J Exp Med. 2012;209(10):1743-52.

17.	Mercadante AA, Dimri M, Mohiuddin SS. Biochemistry, Replication and Transcription. StatPearls. Treasure Island (FL)2021.

18.	Buccitelli C, Selbach M. mRNAs, proteins and the emerging principles of gene expression control. Nat Rev Genet. 2020;21(10):630-44.
