# Supplementary material for: Abnormal expression and the significant prognostic value of aquaporins in clear cell renal cell carcinoma
Source: PLoS One. 2022 Mar 4;17(3):e0264553. doi: 10.1371/journal.pone.0264553 (PMC8896691; doi:10.1371/journal.pone.0264553)
Supplement: Attachment — Submitted filename: Response to reviewers.docx [file pone.0264553.s002.docx]

Dear Editors and Reviewers,

Thanks very much for taking your time to review our manuscript "Abnormal expression and the significant prognostic value of aquaporins in clear cell renal cell carcinoma" (Manuscript Number: PONE-D-21-25893R1). We really appreciate all your comments and suggestions! Your suggestions have enabled us to improve our work. We have resubmitted the file of the revised manuscript with highlighted changes. We have also responded to the comments raised by the reviewers in a point-to-point response manner in this letter. We would like also to thank you for allowing us to resubmit a revised copy of the manuscript.

**Editor comment**

**Comment:** Specifically, reviewer 2 still suggests improvements and the questions raised by reviewer 1 were not fully answered or elucidated. I recommend you address all the points in detail and insert the discussed topics into the manuscript to clarify the interpretation and significance of your data. You should indicate all the modifications in the rebuttal letter.

**Answer:** Many thanks for the editor’ s constructive and professional comments. We have carefully reviewed the relevant literature according to two reviewers’ comments and revised the manuscript accordingly. Particular attention was fixed on interpretation and significance of our findings. Additionally, we added the limitations of our study in the revised manuscript. For details, please refer to the replies to the two reviewers. We sincerely hope that the revised manuscript would meet *PLOS ONE*’s publication criteria.

**Reviewers' comments**

**Reviewer #1:**

**Comment:** Although research project in this study is interesting, I feel that the results have not yet been sufficiently analyzed, or integrated to achieve clear and convincing conclusions. Moreover, any cellular, molecular, and biological experiments were not done to support the author's tentative conclusions. As an example, I concerned about the differences in the expression of AQPs between normal human kidneys and ccRCC. Specifically, I asked about the expression of AQP9, 10, 12A in the kidneys, and the authors should test and compare the expression levels of these AQPs by simple approaches, e.g, PCR or protein experiments. In addition, AQP0, 5, 8 should also be tested since these AQPs are not present in normal kidneys. Thus, I think that the significance and interpretations out of the authors' observations are not clear, and the main points being made are not conclusive.

**Answer:** Very appreciate for your constructive comments. We are very sorry that we did not adequately answer your questions in our last response letter. We have re-reviewed the previous comments and combine them with the comments this time, and have learned that your focus is on the expression level of AQP0/5/8/9/10/12A in the normal kidney tissue. We are very sorry that due to the recent repeated or even exacerbated COVID-19 epidemic in Xi'an, China, our laboratory cannot be opened normally for the time being, so we are afraid we cannot verify the expression level of AQPs in the normal kidney tissue by molecular biology. Therefore, we actively try to solve this problem using alternatives. Genotype-tissue expression (GTEx) project is one of the largest resources, containing about 11,600 RNA-sequenced tissue profiles from over 40 human tissues. We downloaded gene expression and annotation files from the GTEx database and visualized the expression of AQPs in the normal human tissues by R language. As is shown in S1 Fig (see in the next page), AQP0/5/8/9/10/12A are all expressed in the normal kidney tissue to varying degrees although some of them have low expression levels. At the same time, combined with the result in the UALCAN database, we believe that AQPs are expressed in the normal kidney tissue at the transcriptional level (Fig 2). We hope that, to a certain extent, these results could eliminate your confusion about the expression of AQPs in the normal kidney tissue as well as make our results more convincing. We are truly sorry for our inability to conduct the experiment in the short term. We are also aware of this deficiency so we have demonstrated it as the "limitation" in the revised manuscript. And we intend to further explore the expression and molecular mechanism of the members of AQPs in the normal kidney tissue and ccRCC by molecular biological methods when conditions permit in the future.

**S1 Fig. Expressions of AQP0/5/8/9/10/12A in the normal kidney tissue (GTEx).** The GTEx database provides information on 104 normal kidney tissue samples, and the word "kidney" has been bolded and highlighted in red in order to emphasize the expressions of AQP0/5/8/9/10/12A in the normal kidney tissue. The result of GTEx supplements the expression levels of AQPs in the normal kidney to some extent.

**Fig 2. Expressions of AQPs in ccRCC and adjacent normal kidney tissues (Ualcan).**

**Supplements in the text (limitations):**

Our study provides a comprehensive analysis of AQPs in ccRCC using multiple online databases based on the most popular bioinformatics theories. These available methods are characterized by large sample size, low cost, and the ability to perform large-scale genomics studies and functional analyses. However, our study still has some limitations. First, online databases have limitations. Different databases may yield different results due to differences in the sources of the collected samples. Furthermore, this study only presents the results of bioinformatics analysis based on different online databases. Therefore, further molecular biology experiments, including quantitative real time polymerase chain reaction (qRT-PCR), Western blot, and immunohistochemistry, are required to validate the results of this study.

**Reviewer #2:**

**Comments 1:** Table 1. The "Ref" column is still not clearly utilized. List reference numbers for the published papers instead of "name_Renal".

**Answer 1:** It’s a very meaningful comment. Thanks to your guidance, we have made corresponding modifications in Table 1. The revised table is shown below.

**Changes in the table:**

**Table 1. Significant changes of AQPs expression in transcription level between ccRCC and normal kidney tissues (Oncomine).**

|  | **Types of ccRCC VS. Kidney** | **Fold Change** | **p-value** | **t-test** | **Ref** |
| --- | --- | --- | --- | --- | --- |
| AQP1 |  |  |  |  |  |
|  | Non-Hereditary Clear Cell Renal Cell Carcinoma | -4.503 | 1.70E-05 | -4.751 | Beroukhim Renal [23] |
| AQP2 |  |  |  |  |  |
|  | Clear Cell Renal Cell Carcinoma | -5.590 | 2.93E-11 | -13.694 | Gumz Renal [24] |
|  | Clear Cell Renal Cell Carcinoma | -2.567 | 5.55E-6 | -6.779 | Lenburg Renal [25] |
|  | Clear Cell Renal Cell Carcinoma | -13.183 | 0.002 | -5.550 | Yusenko Renal [26] |
|  | Clear Cell Renal Cell Carcinoma | -4.592 | 1.46E-10 | -10.233 | Jones Renal [27] |
|  | Non-Hereditary Clear Cell Renal Cell Carcinoma | -11.212 | 1.87E-08 | -12.468 | Beroukhim Renal [23] |
|  | Hereditary Clear Cell Renal Cell Carcinoma | -12.336 | 4.06E-08 | -13.460 | Beroukhim Renal [23] |
| AQP3 |  |  |  |  |  |
|  | Clear Cell Renal Cell Carcinoma | -3.889 | 4.56E-6 | -7.538 | Higgins Renal [28] |
|  | Non-Hereditary Clear Cell Renal Cell Carcinoma | -3.699 | 8.93E-10 | -8.449 | Beroukhim Renal [23] |
|  | Hereditary Clear Cell Renal Cell Carcinoma | -2.391 | 9.75E-07 | -5.931 | Beroukhim Renal [23] |
| AQP6 |  |  |  |  |  |
|  | Clear Cell Renal Cell Carcinoma | -4.140 | 1.18E-11 | -14.586 | Gumz Renal [24] |
|  | Clear Cell Renal Cell Carcinoma | -8.330 | 1.01E-4 | -7.220 | Yusenko Renal [26] |
| AQP7 |  |  |  |  |  |
|  | Clear Cell Renal Cell Carcinoma | -9.610 | 4.45E-07 | -9.447 | Gumz Renal [24] |
|  | Clear Cell Renal Cell Carcinoma | -2.102 | 3.26E-04 | -4.863 | Lenburg Renal [25] |
| AQP8 |  |  |  |  |  |
|  | Clear Cell Renal Cell Carcinoma | -3.097 | 2.52E-30 | -29.476 | Jones Renal [27] |

**Comments 2:** In the Fig 1 legend, the term "Differences in transcriptional expression" is vague. Precisely define the numbers shown in the cells, and the meanings of the fill colors.

**Answer 2:** It’s a very constructive comment. We have supplemented the specific meaning of the numbers and colors in Fig 1. The revised Fig 1 caption is described as follow.

**Changes in the figure caption:**

**Fig 1. Transcriptional expression of AQPs in 20 different types of cancer diseases (Oncomine).** The numbers in the small boxes represent the number of datasets with statistically significant mRNA differences of the target genes, in which red represents over-expression and blue represents down-regulated expression. Differences in transcriptional expression between ccRCC and normal kidney tissues were compared by students’ t-test. The cut off of p-value and fold change were as following: p-value: 0.01, fold change: 2, gene rank: 10%, data type: mRNA.

**Comments 3:** Fig 3. It would be wise to double-check whether the antibodies used in the Human Atlas database are capable of distinguishing between isoforms AQP12A and 12B. If available antibodies recognize a common epitope, then showing this analysis as separate sets is somewhat misleading. In that case, perhaps the four images should be combined under one subheading '12 A&B' or similar.

**Answer 3:** Very appreciate for your comments. Your sensitive perception is correct. We re-reviewed the description in the manuscript and repeated the analysis in the HPA database. The antibodies that detected AQP12A and AQP12B are indeed identical. Therefore, we have modified Fig 3 and the figure caption as you suggested. The revised figure and figure caption are as follows.

**Changes in the figure and figure caption:**

**Fig 3. Representative immunohistochemistry images of AQPs in ccRCC and normal kidney tissues (Human Protein Atlas).** (**A, C, D, G - I**) AQP0/2/3/6/8/9 proteins were not expressed in ccRCC tissues, whereas their low, medium and high expressions were observed in normal kidney tissues. (**B, E**) Low protein expressions of AQP1/4 were found in ccRCC tissues, while their medium protein expressions were observed in normal kidney tissues. (**F, J - K**) AQP5/10/12A&B proteins were expressed neither in ccRCC nor normal kidney tissues. Republished from https://www.proteinatlas.org/ under a CC BY license, with permission from inger åhlén, original copyright 2021.

**Comments 4:** Fig 4. The legend needs to include a brief description of the type of plot being used to display the data, and definitions of terms and abbreviations shown in the panel keys.

**Answer 4:** Thanks for your reminding. We have modified the caption of Fig 4 based on your suggestion. The modification is as follow.

**Changes in the figure caption:**

**Fig 4. Relationship between mRNA expression of AQPs and individual cancer stages of ccRCC patients (GEPIA).** The figure was visualized using violin plots. mRNA expression levels of AQP1/2/4/6/7/9 were remarkably correlated with patients' individual cancer stages, while the expression across stages did not significantly differ for AQP0/3/5/8/10/11/12A/12B. The method for differential expression gene analysis was one-way ANOVA, using the pathological stage as the variable for calculating differential expression. The F-value was the test statistic from the F-test. The Pr (>F) was the p-value of the F-test and the p-value < 0.05 was significant.

**Comments 5:** Fig 5. Similar to comments above for Fig 4, more details are needed in the legend to define the data shown in each panel.

**Answer 5:** Many thanks for your comments. We're so sorry about this. We have modified the caption of Fig 5 to make the information clearer. The modification is as follow.

**Changes in the figure caption:**

**Fig 5. Genetic mutations in AQPs and their association with the OS and DFS of ccRCC patients (cBioPortal).** (**A**) The bar plot showed the overall alterations of AQPs in the ccRCC samples. The vertical axis represented the percentage of alterations, and the horizontal axis represented the selected tumor sample dataset (ccRCC). Among them, the green indicated mutation, the red was amplification, the blue was deep deletion, the pink was high mRNA expression, and the gray was multiple alterations. A high mutation rate (33.18%) could be observed in ccRCC patients. (**B**) Based on a query of AQPs, cBioPortal summarized genomic alterations in all queried genes across the ccRCC sample set. Each row represented a gene, and each column represented a tumor sample. Brown squares indicated inframe mutations, green squares were missense mutations, gray squares were truncating mutations, red bars were gene amplifications, blue bars were deep deletions, and gray bars with red borders were high mRNA expressions. Mutation rates of each gene were 0%, 5%, 2.5%, 1.8%, 3%, 2.9%, 0.7%, 6%, 0.9%, 4%, 1.1%, 2.7%, 6% and 7%, respectively. (**C**) The figure showed the correlation between AQPs in ccRCC. For each queried gene, Pearson’s correlation coefficients were calculated and displayed in the corresponding boxes. (**D** - **E**) The figure indicated the survival difference between altered and unaltered groups. Genetic alterations of AQPs were not associated with the OS or DFS of ccRCC patients.

**Comments 6:** Text in the Results for Fig 6 would benefit from adding an interpretive summary statement at the end of this section that captures the main outcome or major finding that has been shown as a result of the multiple analyses.

**Answer 6:** Thanks for your suggestions and guidance. We are aware of the lack of summative descriptions and explanations for the text of Fig 6. Therefore, we have made corresponding supplements as follows.

**Supplements in the figure text:**

Overall, AQPs played a significant role in the assembly, trafficking and regulation of gap junctions between cells. Through this special intercellular connection, AQPs regulate the intercellular transport of water, glycerol, carbon dioxide and other substances, thereby maintaining the normal physiological state of cells.

**Comments 7:** In the results text for Fig 7, the extensive numerical details could be moved to a table, and the text focused more clearly on explaining the data shown, with an interpretive summary.

**Answer 7:** Thanks a lot for your valuable suggestions. We have realized that the text for Fig 7 is too cumbersome and lacks of focus. Therefore, based on your suggestions, we have made a table to fill in numerical details, and explained the data shown in detail in the text. We are so sorry that our descriptions of the figures were often not rigorous enough, so we also supplemented the caption of the Fig 7 and corresponding text content with more descriptive and conclusive interpretations. Since the new table is located between the previous Table 1 and Table 2, we have rearranged the order of the tables in the revised manuscript. The modifications are as follows.

**Supplements in the table:**

**Table 2. The correlations of AQPs expression with immune infiltration level in ccRCC.**

| **genes** | **B cells** | **CD8+T cells** | **CD4+T cells** | **macrophages** | **neutrophils** | **dendritic cells** |
| --- | --- | --- | --- | --- | --- | --- |
| AQP0 | 0.057 | -0.04 | 0.038 | 0.025 | 0.087 | 0.02 |
| AQP1 | 0.027 | **0.215** | 0.065 | 0.062 | 0.063 | 0.051 |
| AQP2 | -0.025 | -0.049 | 0.002 | -0.053 | **-0.148** | -0.03 |
| AQP3 | **0.187** | **0.137** | 0.045 | **0.164** | **0.121** | **0.097** |
| AQP4 | -0.029 | **0.116** | **0.101** | **0.197** | **0.176** | 0.057 |
| AQP5 | **-0.147** | -0.052 | **0.124** | -0.048 | -0.083 | -0.078 |
| AQP6 | **-0.111** | **-0.146** | -0.037 | **-0.111** | **-0.128** | **-0.126** |
| AQP7 | -0.072 | -0.029 | -0.067 | -0.072 | **-0.112** | **-0.101** |
| AQP8 | **-0.158** | -0.062 | **0.101** | -0.03 | 0.038 | -0.069 |
| AQP9 | **0.113** | -0.016 | 0.032 | **0.269** | **0.29** | **0.166** |
| AQP10 | -0.053 | **0.169** | **0.166** | **0.11** | **0.112** | 0.062 |
| AQP11 | **0.323** | 0.008 | 0.009 | **0.171** | **0.109** | **0.16** |
| AQP12A | 0.019 | 0.033 | 0.009 | 0.05 | 0.007 | 0.034 |
| AQP12B | 0.03 | 0.044 | 0.073 | **0.097** | 0.057 | 0.082 |

The table showed the associations between individual AQP genes and different immune infiltration by listing the corrected spearman correlation coefficient. The bold values indicated statistical significance (p-value < 0.05).

**Changes in the figure caption:**

**Fig 7. Correlations of B cells, CD8+T cells, CD4+T cells, macrophages, neutrophils, dendritic cells and AQPs in ccRCC (TIMER).** Correlations of mRNA expression levels between cell markers of B cells, CD8+T cells, CD4+T cells, macrophages, neutrophils, dendritic cells and AQPs in ccRCC patients were shown. The ‘partial cor’ indicated the purity-corrected partial Spearman’s rho value, and the ‘p’ referred to ‘p-value’ (p-value < 0.05 was significant).

**Changes in the text:**

Considering that inflammation and infiltrating immune cells would affect the prognosis of ccRCC, we used the TIMER database to comprehensively study the correlation between differentially expressed AQPs and the infiltration of immune cells (Fig 7 and Table 2). It could be found that not every gene of AQPs was closely related to the immune cell infiltration. Individuals with significant immune infiltration correlations were AQP3/4/6/9/10/11. There were also differences in the expression levels of mRNA and protein of these genes in ccRCC, which might mean that there was a certain relationship among the abnormal expression levels of AQPs and the resulting inflammatory microenvironment as well as tumor progression. At the same time, we could observe that AQP3/4/9/10/11 were mainly positively correlated with each immune cell, while AQP6 was negatively correlated with the infiltration degree of immune cells, which suggested that the effects of different AQPs in ccRCC might not be completely consistent. Next, it could be noticed that each gene of AQPs was primarily associated with the infiltration of B cells, macrophages and neutrophils, which indicated that these three immune cells might played a major role in the progression of ccRCC.

**Comments 8:** Results for Fig 8 are said to be aimed at "analyzing AQPs as a whole", but the justification for this is opaque. The Figure seems unnecessary given the extended details provided in the subsequent Fig 9.

**Answer 8:** Many thanks for your recommendation. Your suggestion is correct. The prognostic correlation of overall AQPs shown in Fig 8 is indeed less meaningful than the prognostic status of individual genes illustrated in Fig 9 . Therefore, we decided to delete the Fig 8 and modify the serial numbers of Fig 9 and Fig10 (become the new Fig 8 and Fig 9).

**Comments 9:** Figs 9 and 10. Using the same headings "overall survival" or "disease free survival" for all panels is not informative. The legend could state what type of survival is plotted, and the panel headings then more helpfully could list the AQP class being studied.

**Answer 9:** Thank you for your professional direction. We have redrawn the Fig 9 and Fig 10 (since we have removed the previous Fig 8, their new serial numbers are Fig 8 and Fig 9). The modifications are as follows.

**Changes in the figures:**

 **Fig 8. Prognostic significance of AQPs for the OS of ccRCC patients (GEPIA).**

 **Fig 9. Prognostic significance of AQPs for the DFS of ccRCC patients (GEPIA).**

**Comments 10:** The data appear to show that AQP1 levels are the most important predictor of survival, as compared to relatively smaller effects of other AQPs, but this idea (if correct) is not clearly evaluated as a possible principal outcome of the study in the Discussion or Conclusions.

**Answer 10:** Thanks for your suggestions. Your idea is correct. Through our multiple analyses and comprehensive consideration, AQP1 does play a significant role in ccRCC and should be further elaborated. At the same time, we also would like to supplement the explanation of AQP9 in the manuscript, because AQP9 showed equally significant effects, whether in expression level, gene mutation status, immune cell infiltration, or prognostic relevance. Therefore, we reviewed the relevant literature and further elaborated on AQP1/9 in the Discussion and also summarized it in the Conclusions. The changes are as follows.

**Supplements in the text (the Discussion):**

Finally, it is worthy to pay special attention to the independent prognostic factors AQP1 and AQP9 that are finally screened. Through multi-dimensional analysis, it is not difficult to find that there are significant changes in the mRNA expression levels of AQP1 and AQP9 in ccRCC, and both will change significantly with the progression of ccRCC stages. It is worth noting, however, that AQP1 and AQP9 have different trends in these respects. The expression level of AQP1 in ccRCC shows a gradually decreasing trend, while that of AQP9 is the opposite. In terms of mRNA mutation and immune cell infiltration, the expressions of AQP1 and AQP9 are relatively consistent. The mRNA expression levels of AQP1 and AQP9 are positively correlated with the infiltration of most immune cells. Clinical prognosis correlation analysis indicated that the group with a high expression level of AQP1 tends to show better OS and DFS, while the group with a high expression level of AQP9 means poorer OS and DFS. It is well known that the occurrence of most kidney cancers is closely related to the abnormal function of the von Hippel-Lindau (VHL) gene. The abnormality of the VHL gene can lead to the reduction and accumulation of hypoxia-inducible factor (HIF), thereby initiating the transcriptional activation of hypoxia-responsive genes, and ultimately promoting the occurrence and development of cancer [76]. In the pathway AQP1 is involved in, the up-regulation of AQP1 can promote the stabilization of HIF, which in turn makes the high expression group show a better prognosis [33]. Jing JB et al. explored the expression of AQP9 which was consistent with ours, and they also analyzed the relationship between AQP9 and tumor environment. The results showed that in ccRCC, AQP9 promoted tumor-associated macrophage polarization, inhibited the recruitment of natural killer (NK) cells and CD8+ T cells by inhibiting P53 and activating the Janus kinase/signal transducer and activator of transcription (JAK/STAT) pathway, and ultimately stimulated a reorientation of the tumor microenvironment in ccRCC toward tumor-friendly directions [56]. Combining the above analyses of AQP1 and AQP9, we may guess that AQP1 is the tumor suppressor gene of ccRCC while AQP9 is the proto-oncogene of ccRCC. Although both genes belong to the AQPs family, they may play diametrically opposite roles, which are interesting and worthy of further exploration.

**Changes in the text (the Conclusions):**

In this study, the Oncomine database and the UALCAN database were used to analyze the mRNA of 14 members of AQPs, and the prognostic values of AQPs in ccRCC were analyzed using Kaplan-Meier plotter and GEPIA database. The results showed that in ccRCC patients, the mRNA expression levels of AQP0/8/9/10 were up-regulated in ccRCC, while those of AQP1/2/3/4/5/6/7/11 were down-regulated in ccRCC. The clinical database showed that the high mRNA expression levels of AQP0/8/9 were significantly associated with poor OS. On the contrary, the high levels of AQP1/2/3/4/5/6/7/10/11, especially the high levels of AQP1/4/7 were correlated with better OS in ccRCC patients. Among them, AQP1/7/11 have similarities in the tissue structure and positioning, and they are all highly expressed in the proximal tubules of the kidney. Perhaps AQP7/11 can become a potential diagnostic index and therapeutic target for ccRCC after AQP1. As the only two independent prognostic factors, AQP1 and AQP9 have shown differential expressions in ccRCC and normal kidney tissues as well as significant immune cell infiltration, which indicates that AQP1 and AQP9 may be used as a new prognostic and diagnostic marker in ccRCC. At the same time, each AQPs member may exert its function through different signaling pathways. To further explore the role of AQPs in ccRCC, more refined mechanism research and big data clinical trials are needed. Our study comprehensively analyzed the transcriptomics characteristics and the prognosis value of AQPs in ccRCC. Based on the verification of a large amount of data, the members of AQPs are expected to become new diagnostic, prognostic or therapeutic biomarkers markers for ccRCC targeted therapy.

**Comments 11:** Minor grammar errors throughout could use polishing by a language-fluent editor to showcase the work as effectively as possible, but it is understandable as written.

**Answer 11:** We sincerely appreciate your tolerance and understanding. We have used language editing software to check for grammatical errors that still exist in the manuscript and have made appropriate revisions.

**References:**

23.	Beroukhim R, Brunet JP, Di Napoli A, Mertz KD, Seeley A, Pires MM, et al. Patterns of gene expression and copy-number alterations in von-hippel lindau disease-associated and sporadic clear cell carcinoma of the kidney. Cancer Res. 2009;69(11):4674-81.

24.	Gumz ML, Zou H, Kreinest PA, Childs AC, Belmonte LS, LeGrand SN, et al. Secreted frizzled-related protein 1 loss contributes to tumor phenotype of clear cell renal cell carcinoma. Clin Cancer Res. 2007;13(16):4740-9.

25.	Lenburg ME, Liou LS, Gerry NP, Frampton GM, Cohen HT, Christman MF. Previously unidentified changes in renal cell carcinoma gene expression identified by parametric analysis of microarray data. BMC Cancer. 2003;3:31.

26.	Yusenko MV, Kuiper RP, Boethe T, Ljungberg B, van Kessel AG, Kovacs G. High-resolution DNA copy number and gene expression analyses distinguish chromophobe renal cell carcinomas and renal oncocytomas. BMC Cancer. 2009;9:152.

27.	Jones J, Otu H, Spentzos D, Kolia S, Inan M, Beecken WD, et al. Gene signatures of progression and metastasis in renal cell cancer. Clin Cancer Res. 2005;11(16):5730-9.

28.	Higgins JP, Shinghal R, Gill H, Reese JH, Terris M, Cohen RJ, et al. Gene expression patterns in renal cell carcinoma assessed by complementary DNA microarray. Am J Pathol. 2003;162(3):925-32.

33.	Huang Y, Murakami T, Sano F, Kondo K, Nakaigawa N, Kishida T, et al. Expression of aquaporin 1 in primary renal tumors: a prognostic indicator for clear-cell renal cell carcinoma. Eur Urol. 2009;56(4):690-8.

56.	Jing J, Sun J, Wu Y, Zhang N, Liu C, Chen S, et al. AQP9 Is a Prognostic Factor for Kidney Cancer and a Promising Indicator for M2 TAM Polarization and CD8+ T-Cell Recruitment. Front Oncol. 2021;11:770565.

76.	Maynard MA, Ohh M. Von Hippel-Lindau tumor suppressor protein and hypoxia-inducible factor in kidney cancer. Am J Nephrol. 2004;24(1):1-13.
